# CRISPR-Cas9, a tool to efficiently increase the development of recombinant African swine fever viruses

**DOI:** 10.1038/s41598-018-21575-8

**Published:** 2018-02-16

**Authors:** Manuel V. Borca, Lauren G. Holinka, Keith A. Berggren, Douglas P. Gladue

**Affiliations:** 1 Agricultural Research Service (ARS), Plum Island Animal Disease Center, Greenport, NY 11944 USA; 2 Oak Ridge Institute for Science and Education (ORISE), Oak Ridge, TN 37831 USA

## Abstract

African swine fever virus (ASFV) causes a highly contagious disease called African swine fever. This disease is often lethal for domestic pigs, causing extensive losses for the swine industry. ASFV is a large and complex double stranded DNA virus. Currently there is no commercially available treatment or vaccine to prevent this devastating disease. Development of recombinant ASFV for producing live-attenuated vaccines or studying the involvement of specific genes in virus virulence has relied on the relatively rare event of homologous recombination in primary swine macrophages, causing difficulty to purify the recombinant virus from the wild-type parental ASFV. Here we present the use of the CRISPR-Cas9 gene editing system as a more robust and efficient system to produce recombinant ASFVs. Using CRISPR-Cas9 a recombinant virus was efficiently developed by deleting the non-essential gene 8-DR from the genome of the highly virulent field strain Georgia07 using swine macrophages as cell substrate.

## Introduction

The virus family Asfarviridae consists of only one virus, African swine fever virus (ASFV), the etiological agent that causes African swine fever (ASF). This large double stranded DNA virus has more than 150 ORFs that are encoded in the 180–190 kilobase genome. ASF disease, can range from sub-clinical to lethal depending both on the specific host that is infected and the specific strain of virus^1^. Several sub-Saharan African countries and Sardinia (Italy) have endemic ASF. Recent outbreaks of ASFV started with only a single introduction of ASFV in the Caucasus region in 2007. This outbreak affected Georgia, Armenia, Azerbaijan and Russia and more recently has spread as far west as Warsaw, Poland causing the fear that this disease could disseminate into other neighbouring European countries^2^. The concern is due to the current outbreak strain has the ability to be highly contagious and in domestic pigs it often causes lethality. Due to the potential and wide spread loss of domestic pigs, the swine industry could suffer from substantial economic consequences should an outbreak occur^3^.

Developing recombinant ASFV from wild type field isolates is necessary to gain basic knowledge regarding the role of specific genes in different processes of virus replication, virus-host interaction or virus virulence. The ability to develop recombinant viruses from wild type field isolates, which only replicate in primary swine macrophages cultures, has been extremely difficult with only a few successful attempts^4–6^. Similarly, the development of the only reported experimental live-attenuated ASF vaccines, have also relied on the production of recombinant field isolates by genetic manipulation, in which one or more genes were deleted from the field isolate^7–9^.

Adaptation of ASFV to stable cell lines has caused spontaneous deletions to substantial areas of the viral genome, often leading to loss of virus virulence, but not always gaining the ability of offering protection against homologous virulent parental challenge^10^. Therefore, in order to keep the viral genome stable, recombinant ASFV have to be produced in primary cell cultures of swine macrophages. Production of recombinant ASFV in such cell cultures have been shown to be methodologically challenging. Construction of recombinant ASFV has typically relied on performing homologous recombination by introducing a plasmid containing homologous arms of approximately 1000 bp to ASFV from both sides of the targeted gene, and replacing the targeted gene with a reporter gene in the plasmid^5,11^. This homologous recombination procedure, particularly performed in swine macrophage cultures, has a very low level of efficiency in producing a recombinant virus as it relies on the rare and random occurrence of homologous recombination between the viral DNA and the introduced plasmid DNA. This results in a high background of wildtype virus making purification of recombinant ASFV difficult, requiring the repetition of many rounds of methodologies as plaque purification or limiting dilution to achieve the purification of a recombinant ASFV.

The Clustered Regularly Interspaced Short Palindromic Repeats (CRISPR)/CRISPR-associated nuclease 9 (Cas9) system was originally discovered as a natural microbial immune mechanism to protect against invading viruses or other genetic elements^12–14^. Adaptation of this pathway to achieve effective genome editing using site specific DNA double strand breaks allowed for the natural CRISPR system to specifically edit mammalian genomes with a very high degree of accuracy^15^. CRISPR-Cas9 uses a 20 nucleotide targeting sequence inside of its directed RNA, called a short guide RNA (gRNA), to target the location in the genome for editing. Several viruses such as type I herpes simplex virus^16^, Epstein-Barr virus^17^, Pseudorabies virus^18^ and Vaccinia virus^19^ have been edited using this system.

This report describes for the first time the successful gene editing of ASFV using the CRISPR-Cas9 system by removing the ASFV gene *8-DR* that encodes for a gene previously described as having some similarity to swine CD2 gene^20^ and replacing it with the red fluorescent protein (RFP) gene. ASFV gene *8-DR* has previously been deleted using traditional homologous recombination and has been determined to be non-essential. The deletion of 8-DR is easily observed by the loss of ability in the resulting virus to hemadsorb swine red cells, preventing rosette formation^20,21^. Results presented here demonstrate that use of the CRISPR-Cas9 system may represent a significant increase in the efficiency for developing recombinant ASFVs.

## Results and Discussion

**Figure 1:**
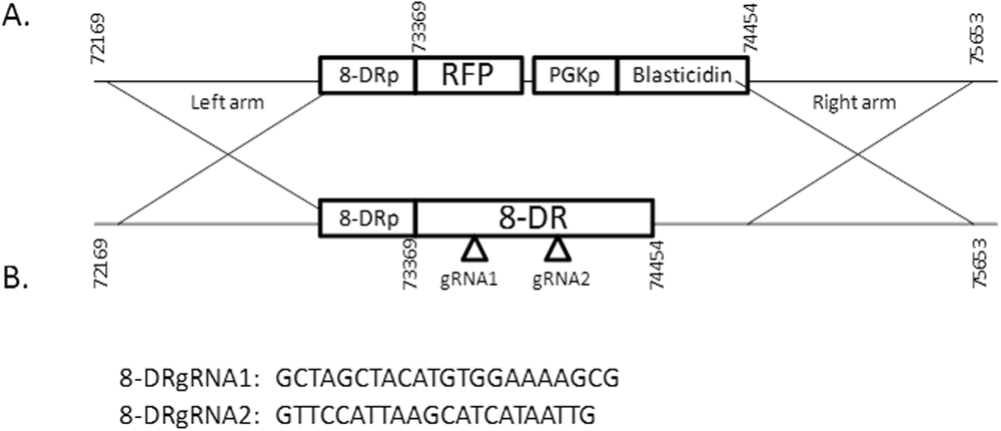
(**A**) Schematic representation of the 8DR gene deleted in ASFV-GΔ8-DR.CR and replaced with the RFP and blasticidin reporter gene cassette. (**B**) Sequence of the 8DR targeting gRNAs used to target the ASFV genome for editing.

The CRISPR-Cas9 methodology used in this study induces a Cas9 double stranded break into the ORF of 8-DR in the ASFV genome, and the replacement of the 8-DR ORF with the insertion cassette containing the selectable marker red fluorescent protein (RFP). The donor plasmid is depicted in Fig. 1A, and was used as a template for replacing the ORF of 8-DR and contains homologous arms surrounding the replacement cassette containing RFP. The left arm contains the region preceding the 8-DR open reading frame from position 72,169 to 73,369 in the ASFV-G genome and the right arm contains the region that proceeds the 8-DR open reading frame from position 74,454 to 75,653 in the ASFV-G genome. This design allows for the CRISPR-Cas9 guided homologous recombination to occur specifically, replacing the ORF of 8-DR with RFP and Blasticidin genes, allowing RFP to be under the control of the natural 8-DR promoter.

The two gRNAs were designed using the Blue Heron Biotech (Bothell, WA), Guide RNA Target Design tool available at https://wwws.blueheronbio.com/secure/tools/gRNASrc.jsp. The sequences of the gRNAs used are described in Fig. 1B, and were inserted into the pCAS-Guide vector (Blue Heron Biotech) which contains a *Cas9* gene under the control of a CMV promoter, and our target gRNA along with the gRNA scaffold required to perform the double stranded break under the control of a U6 promoter. Our target gRNAs align to regions in the ORF of 8-DR, specifically positions 73,528 to 73,547 (8-DR gRNA1) and positions 73,991 to 74,010 (8-DR gRNA2).

**Figure 2:**
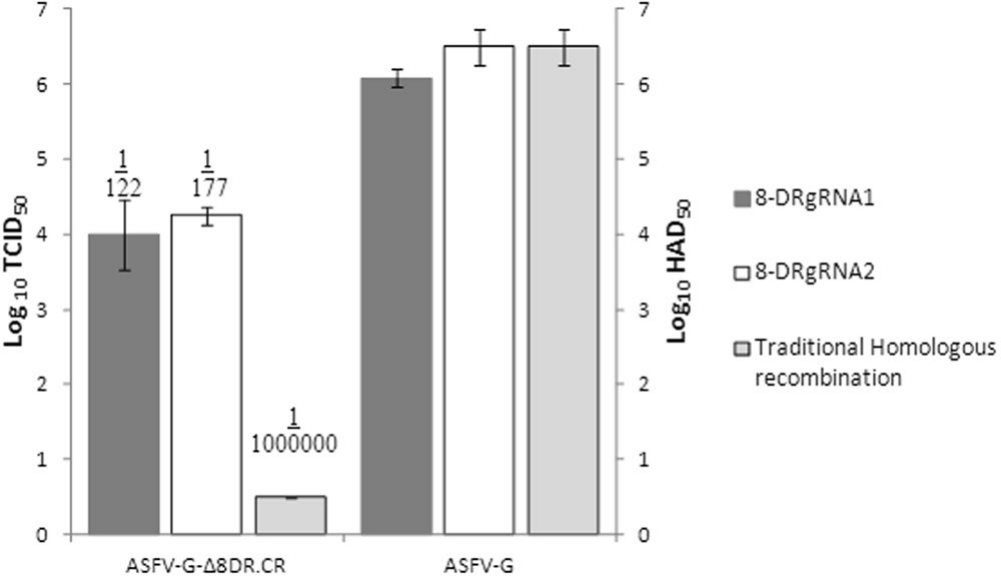
Comparison of recombination efficiency of the two 8DR targeting gRNAs and traditional homologous recombination either by hemadsorption or by detection of RFP expressed as log_10_ HAD_50_/ml or log_10_ TCID_50_/ml. Frequency of recombination obtained with either technique is expressed as the ratio between the titers of the recombinant and the parental viruses.

The efficiency of the CRISPR/Cas9 system using two different targeting gRNAs for 8-DR were compared to traditional homologous recombination techniques, in both cases substituting the *8-DR* gene for the *RFP* gene. Primary swine macrophage cell cultures were made and seeded as previously described^22^, and infected with ASFV-G at an MOI of 10 for 4 hours at 37 °C under 5% CO_2_. The inoculum was then removed. After infection, as indicated in Fig. 2, cells were either co-transfected with the donor plasmid and the indicated targeting gRNA for 8DR or, alternatively, transfected with the homologous recombination plasmid. All transfections were performed using Fugene HD following the manufacturer’s protocol. After 24 hours, the plates were frozen, and the cell culture supernatants collected.

To determine the efficiency of Cas9 to edit the ASFV genome, titrations of the harvested virus were performed on day 5 by evaluating for HA or for the presence of fluorescence as described in the materials and methods. Cells infected with 8-DR-negative recombinant virus (ASFV-G-Δ8-DR.CR) were characterized by presence of fluorescence and absence of HA while cells infected with parental wild type virus display the absence of fluorescence and presence of HA. The Reed and Muench method was used for titer calculation^23^ and the presence of HA (HAD_50_) or for the presence of fluorescence (TCID_50_) was determined (Fig. 2) we have previously determined that titers observed by fluorescence are equal to titers observed by HA^24^. We observed that under these conditions detection of recombinant viruses obtained by traditional homologous recombination techniques resulted in being below our limit of detection for titration (<0.5 Log_10_TCID_50_), but recombinant virus expressing fluorescence was still able to be recovered by blind passaging. However, using the CRISPR/Cas9 system (regardless the targeting gRNA utilized) approximately 4 logs of recombinant ASFV were noticeably detected at 24 hours post-transfection. These results show a clear advantage of producing recombinant ASFV using CRISPR/Cas9 over traditional homologous recombination techniques.

Frequency of recombination obtained with the CRISPR/Cas9 system is expressed as the ratio between the titers of the recombinant and the parental viruses, resulting in 1 recombinant virus for every 122 or 178 wild type viruses for gRNA1 and gRNA2, respectively. This was a significant improvement over the traditional homologous recombination technique which was less than one recombinant virus for every 10^6^ wild type viruses, as the traditional homologous recombination technique was unable to be detected by titration These results clearly demonstrate the potential advantage of using CRISPR/Cas9 over homologues recombination as a methodology to produce recombinant ASFV.

**Figure 3:**
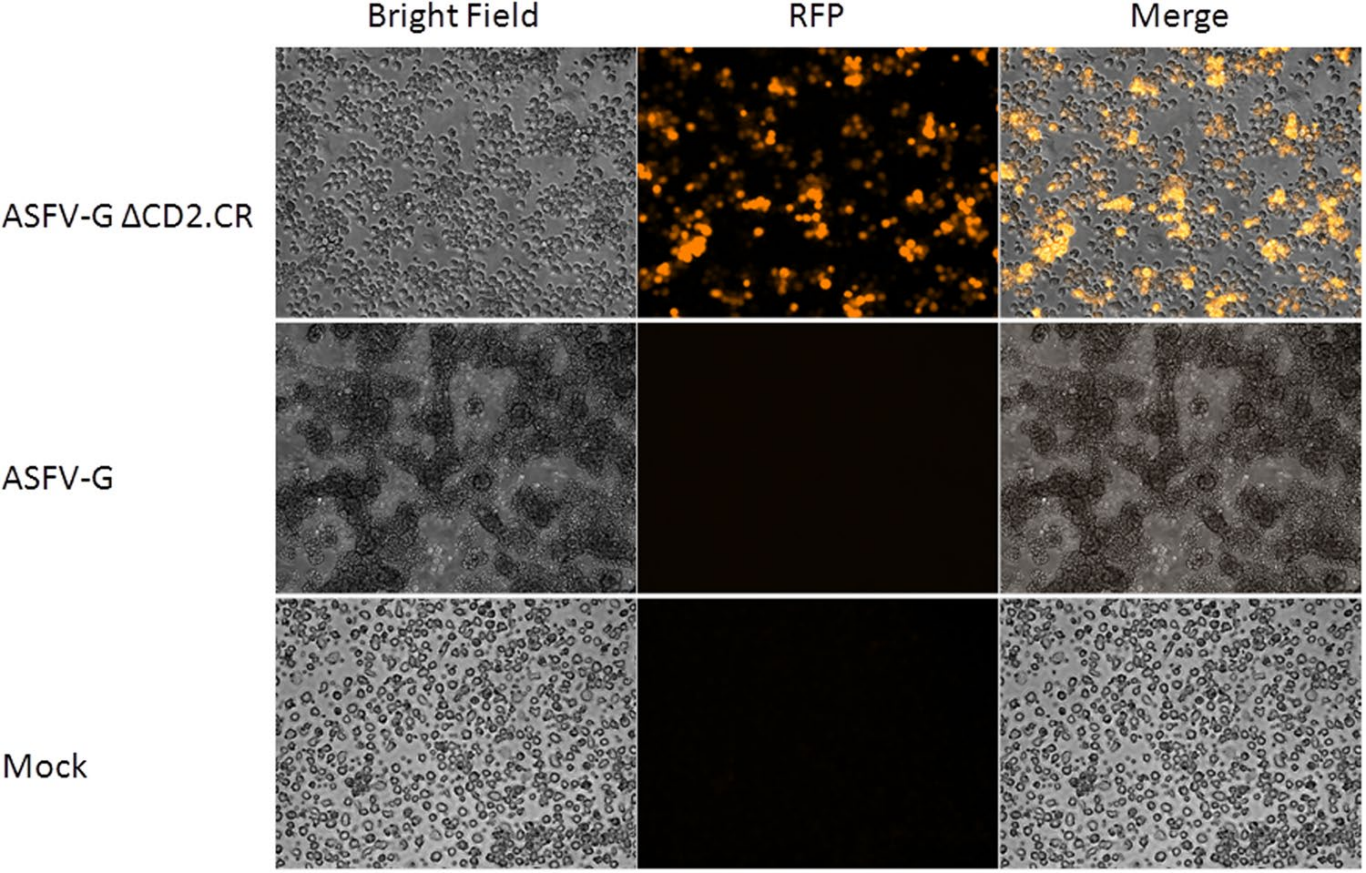
Primary swine macrophages were inoculated with the indicated virus or mock infected, in the presence of red blood cells. In the first column bright field images were taken 20hpi, and observed for hemadsorption. In the second column RFP fluorescence was examined. The two images were merged in the third column.

Purification of the recombinant viruses developed by CRISPR/Cas9, ASFV-G-Δ8-DR.CR, was achieved by using limited dilution. Monitoring of virus purification was made by the presence of fluorescent cells simultaneously with the absence of rosette formation. Cell infected with ASFV-G-Δ8-DR.CR show clear RFP fluorescence and absence of rosette formation whereas cells infected with ASFV-G show clear rosette formation and absence of RFP fluorescence. Mock-infected cells show neither rosette formation nor fluorescence (Fig. 3). As expected, starting with an increased concentration of recombinant virus using the CRISPR/Cas9 system allowed for an easier purification process than using traditional homologous recombination methods with a very low rate of recombination. Purification of ASFV-G-Δ8-DR.CR took 5 cloning steps by limiting dilution, which is a much shorter process than what was needed when we used homologous recombination for other recombinant viruses we have produced in the past^8,9,20,24,25^, usually requiring additional purification steps to obtain pure recombinant virus.

Full genome sequence was used in order to determine that only the specific modification was introduced into the genome of the recombinant virus. ASFV-G-Δ8-DR.CR was sequenced using NGS on the Illumina NextSeq, as previously described^25^. We have previously reported several differences in the full-length genomes of parental ASFV-G with a similar genome sequence ASFV Georgia 2007/1^2^ available on GenBank (#FR682468)^25^. The consensus sequence of the ASFV-GΔ8-DR.CR genome had a 2.3Kb nucleotide insertion replacing the 8-DR ORF corresponding to the insertion cassette in Fig. 1A, being introduced into the location targeted by the arms. Other than the expected reporter cassette, no genetic changes were found in the ASFV-GΔ8-DR genome when compared to the parental ASFV-G genome. In summary, ASFV-G-Δ8-DR.CR did not have unexpected additional mutations from using CRISPR/Cas9 or purifying the recombinant virus.

To summarize, we describe the development of a gene deleted recombinant virus based on the highly virulent ASFV-G for the first time using the CRISPR/Cas9 system. We show that CRISPR/Cas9 modification of the ASFV genome can potentially occur at a much higher frequency than when traditional homologous recombination is used. This finding represents a potential significant advancement in recombinant ASFV technology over traditional homologous recombination techniques which would imply a substantial improvement in the feasibility of developing recombinant ASFV. Previously described gene deletions in ASFV that were determined to be lethal have relied on methods using traditional homologous recombination methods to create recombinant ASFV, where the lack of producing a virus with a gene deletion would lead to the determination of gene deletions being lethal. However, it is possible the low frequency of recombination using traditional homologous recombination methods may have just made purification too difficult. Therefore, some of those previous attempts to delete virus genes considered lethal should be revisited using CRISPR/Cas9 methodology. Although this report significantly increased our ability to achieve a higher recombination frequency, we were still only able to get less than one 1% recombinant virus on the initial transfection, it is possible that the transfection efficiency could be improved to facilitate an increase in recombinant virus in the initial transfection. However, it is possible that the CRISPR/Cas9 will always be unable to edit all the copies of the ASFV genome in a single cell before the viral DNA is packaged.

## Materials and Methods

### Cell cultures and viruses

As previously described^22^ defibrinated swine blood was used to prepare cell cultures of primary swine macrophage. In brief, swine blood was heparin-treated and incubated at 37 °C for 1 hour allowing separation of the erythrocyte fraction. A density gradient of Ficoll-Paque with a 1.079 specific gravity was used with the Mononuclear leukocytes on top. The cell fraction consisting of monocyte/macrophage cells was collected and cultured for tissue culture with macrophage media. Adherent cells were reseeded with a density of 5 × 10^6^ cells/ml in 6-well dishes to conduct the CRISPR/Cas9 experiments or into 96 well plates for titration at 1 × 10^5^/ml and allowed to attach for 24 hours before experimentation.

Virus titrations was performed by making dilutions in macrophage media as previously described^24^ Swine macrophages were plated at 5 × 10^6^ cells per plate in 96-well plates for all virus titrations. Virus titers were assessed every day for 5 days after being plated where on the fifth day final virus titers were determined by using the established Reed and Muench method either by HAD_50_ for the presence of HA or TCID_50_ for the presence of fluorescence.

ASFV-G a field isolate from the Republic of Georgia was provided by Dr. Vepkhvadze, from the Laboratory of the Ministry of Agriculture in Tbilisi^10^.

### Plasmid design for traditional recombination

Plasmid pBluscript II SK (-) lacking its multiple cloning was used as a backbone, the recombination cassette was inserted at the SphI restriction site after the T7 promoter. The recombination cassette contains a left recombination arm that is 1000 bp upstream of ORF 8-DR identical to ASFV-G nucleotide positions 72369–73368, followed by the p72 promoter identical to ASFV-G nucleotide positions on the negative strand 105720–105533, followed by RFP, and a nopaline synthase (nos) termination sequence genbank AB697057.1 nucleotide positions 1870–2192, followed by a right recombination arm that is 1000 bp downstream of 8-DR identical to ASFV-G nucleotide positions 74452–75451.

### CRISPR/Cas9 transfection and frequency calculations

CRISPR/Cas9 experiments were conducted after a 1 hour of virus adsorption at 37 °C and CO_2_ at 5%, the inoculum was then discarded, the indicated plasmids were transfected with Fugene HD following the manufactures protocol (available: http://www.promega.com/techserv/tools/FugeneHdTool/). A 3:1 Fugene:DNA ratio was used with 3.3ug of DNA and 9.9ul of Fugene HD. The complex was mixed carefully by pipetting and incubated for 10 min; 150 ul of the complex was added to the cells dropwise. Cells were then incubated at 37 °C under 5% CO_2_ and observed for presence of RFP and frozen 24 hours post-transfection, thawed and titrated. Frequency of transfection was calculated as the ratio between titters of the recombinant and the parental viruses.

### Complete sequencing of ASFV genomes using Next Generation Sequencing

Macrophage cells were seeded as described and infected with ASFV, once the cytopathic effect was evident throughout the monolayer, DNA was isolated as described previously from cells infected with ASFV^10^. The extracted DNA was then used to completely sequence the virus DNA as previously described^10^. In Brief, the viral DNA was sheared using enzymatic reactions assessed for the distribution of size fragmentation, then ligation of indentifying barcodes using an adapter sequence were added to the DNA fragments. Using a Pippin Prep™ (Sage Science, Beverly, MA) the required size range of the library was collected, and normalized. We then used this DNA library for NGS sequencing using the NextSeq (Illumnia, San Diego,CA) following the manufactures protocol. Sequence analysis was performed using CLC Genomics Workbench software (CLCBio, Waltham, MA).
